# Application of four-dimensional cone beam computed tomography in lung cancer radiotherapy

**DOI:** 10.1186/s13014-023-02259-8

**Published:** 2023-04-17

**Authors:** Muyasha Abulimiti, Xu Yang, Minghui Li, Fukui Huan, Yanxin Zhang, Liang Jun

**Affiliations:** 1 National Cancer Center, National Clinical Research Center for Cancer/Cancer Hospital & Shenzhen Hospital, Chinese Academy of Medical Sciences and Peking Union Medical College, Shenzhen, 518116 China; 2 Department of Radiation Oncology, National Clinical Research Center for Cancer/Cancer Hospital, National Cancer Center, Chinese Academy of Medical Sciences and Peking Union Medical College, Beijing, 100021 China

**Keywords:** 4D cone beam CT, Lung cancer, Radiotherapy

## Abstract

**Objective:**

This study explored the application of four-dimensional cone beam computed tomography (4D CBCT) in lung cancer patients, seeking to improve the accuracy of radiotherapy and to establish a uniform protocol for the application of 4D CBCT in radiotherapy for lung cancer.

**Methods:**

4D CBCT was applied to evaluate tumor volume response (TVR), motion, and center coordinates during radiotherapy in 67 eligible individuals with lung cancer diagnoses. The differences between 4D CBCT and 3D CBCT in different registration methods were compared.

**Results:**

TVR was observed during treatment in 41% of patients (28/67), with a mean volume reduction of 41.7% and a median time to TVR of 19 days. Tumor motion was obvious in 16 patients, with a mean value of 0.52 cm (0.22 to 1.34 cm), and in 3 of 6 tumors close to the diaphragm (0.28 to 0.66 cm). Gray value registration based on mean density projection could still achieve close results to the 4D gray value registration. However, when the registration was based on bone alone, partial off-targeting occurred in the treatment in 41.8% of cases. The off-target rate was 19.0% when the tumor motion was ≤ 0.5 cm and 52.2% when the motion was > 0.5 cm.

**Conclusion:**

Tumor volume and motion of intrapulmonary lesions in individuals diagnosed with lung cancer varied significantly in the third week of radiotherapy. 4D CBCT may be more advantageous for isolated lesions without reference to relative anatomical structures or those near the diaphragm. Grayscale registration based on mean density projection is feasible.

## Introduction

Lung cancer is the most frequently diagnosed cancer worldwide, and the number of lung cancer diagnoses continues to rise, showing a high incidence rate and a low survival rate, with the associated mortality accounting for 19.4% of deaths among all individuals diagnosed with cancer [1]. In the multidisciplinary treatment of lung cancer, radiotherapy is mainly used for stereotactic large-split radiotherapy for early-stage lung cancer, adjuvant or radical treatment is chosen for intermediate stage tumors, and palliative treatment is available for the advanced stage. Stereotactic body radiotherapy (SBRT) is the standard treatment for early inoperable cases [2]. However, uncertainty in the spatial location of the tumor can significantly impact the implementation of SBRT. Furthermore, studies have demonstrated that off-target radiation based solely on population data can lead to inaccurate treatment volumes, which in turn can cause decreased local control rates and increased dose-limiting toxicity in normal tissues [3]. Radiotherapy for lung cancer, therefore, requires strict control of tumor motion to ensure that the tumor is irradiated at an adequate dose and the surrounding normal tissues are maximally protected. Four-dimensional cone beam computed tomography (4D CBCT) is an on-board imaging system that acquires respiratory motion data during the frame rotation, enabling the generation of four-dimensional images before, during, and after treatment [4]. The 4D CBCT images can also be aligned with Four-dimensional computed tomography (4DCT) positioning images to correct setup errors and to monitor the range of tumor motion in real-time, ensuring that the moving tumor is within the irradiation range and facilitating the implementation of precision radiotherapy. The implementation of 4D CBCT overcomes the defect that three-dimensional (3D) CBCT is a quick scan, reflects the one-sided range of tumor motion, and can evaluate the amplitude and range of tumor motion online. However, because of the long scanning time of 4D CBCT and the demanding technical requirements, its use in the clinic has been limited, and no uniform application scheme is recommended. This paper explores the use of 4D CBCT in lung cancer radiotherapy for monitoring tumor volume response (TVR) and tumor motion and attempts to establish a protocol for the use of 4D CBCT in lung cancer radiotherapy.

## Materials and methods

### Patients

1. A total of 67 lung cancer patients who received thoracic radiotherapy in the Cancer Hospital of the Chinese Academy of Medical Sciences from 2015 to 2018 were enrolled. The inclusion criteria were: (1) age ≥ 18 years; (2) Karnofsky Performance Scale (KPS) ≥ 90; (3) able to tolerate long scanning times; (4) visible intrapulmonary lesions.
2. The general clinical characteristics of the enrolled patients are displayed in Table 1.

**Table 1:** Clinical characteristics of study patients (N = 67)

	Group	Number (%)	Mean	Median	Range
Gender	Male	50 (74.6)	-	-	-
	Female	17 (25.4)	-	-	-
Age	36–60	27 (40.3)	-	-	-
	61–70	24 (35.8)	-	-	-
	≥ 71	16 (23.9)	63	63	36–82
BMI (kg/m^2^)	-	-	24.79	24.49	15.85–32.53
Tumor volume	0–50	45 (67.2)	42.31	13.81	0.44–285.78
	50–300	22 (32.8)			
Tumor site	Upper lobe of the right lung	19 (28.4)	-	-	-
	Middle lobe of the right lung	5 (7)			
	Lower lobe of the right lung	17 (25.4)			
	Upper lobe of the left lung	8 (13.4)			
	Lower lobe of the left lung	18 (26.8)			
Adjacent to surrounding structures	Mediastinum	19 (28.4)	-	-	-
	Chest wall	15 (22.4)			
	Diaphragm	8 (11.9)			
Pulmonary atelectasis or obstructive pneumonia	Yes	5 (7.5)	-	-	-
	No	62 (92.5)			
Image datasets	4D CBCT	260	-	-	-
	3D CBCT	228			

### Methods

**Fig. 1:**
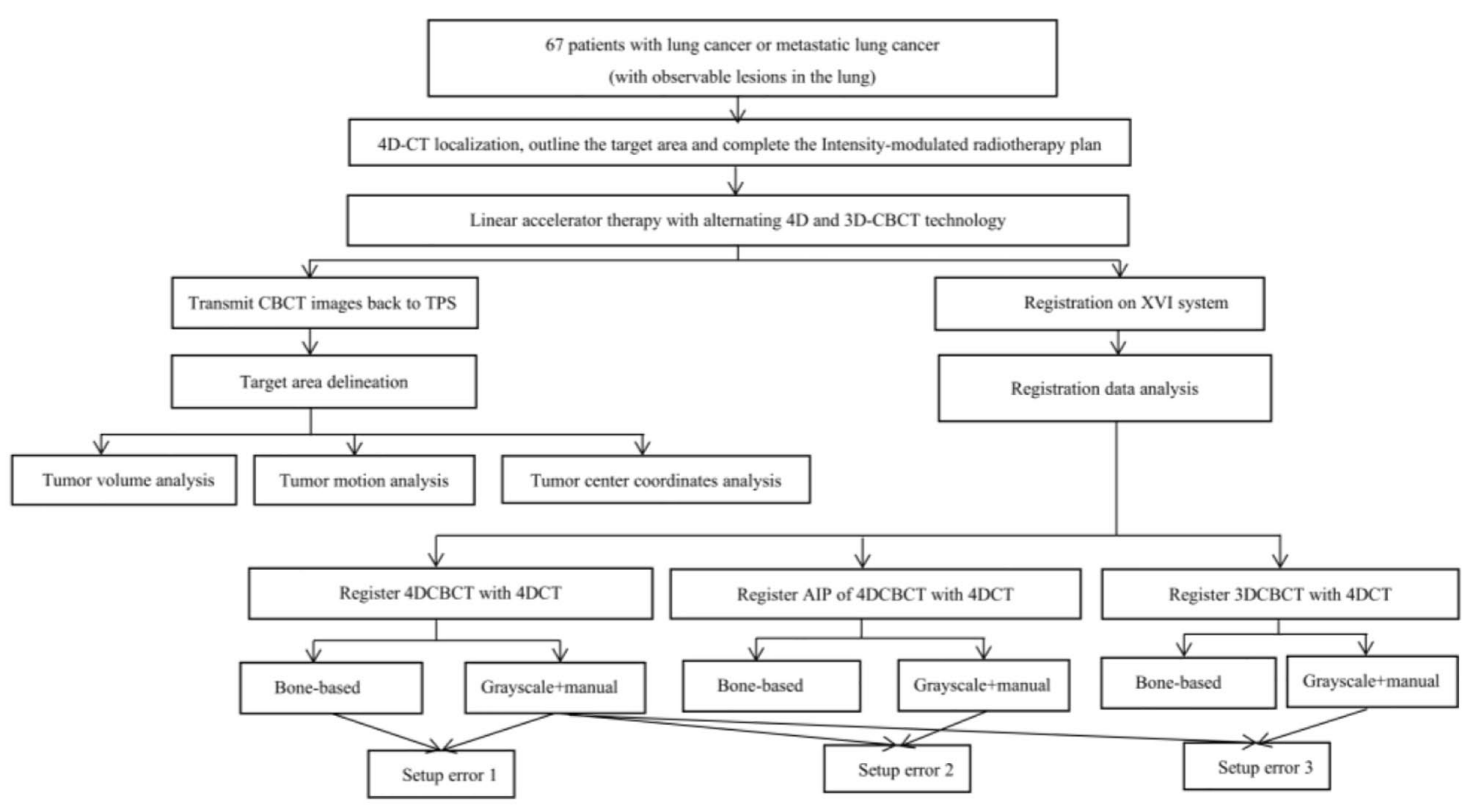
Study flowchart

Localization of all tumors was by 4DCT and image-guided radiotherapy was performed with 4D CBCT after the completion of the treatment plan. At least 3 to 4 CBCT scans were completed in every case. Along with the planning 4DCT data, all 4D CBCT images and setup error data were collected, and a database and image library was established. In Part1: Tumor Volume、Motion and Center coordinates of tumor were compared between 4DCBCT and planning 4DCT, and the clinical value of 4DCBCT was determined by the characteristics of the tumor changes. During comparison of the tumor motion, the position of the tumor in three axes in 4DCBCT in the complete respiratory phase (0–100%) was compared with the position of the tumor in 4DCT, and the point with the greatest difference of activity was selected and compared with the extreme difference of action in each scan to explore the activity pattern. In Part 2: Different images were registrated with 4DCT for comparison of setup errors and to explore the most feasible registration method. SPSS11.5 software was used to calculate the mean offset values and the means ± standard deviations of setup errors according to the data, and a t-test was performed. Setup error1 was compared between 4DCBCT bone-based registration and grayscale + manual registration, Grayscale + manual registration was defined according to gray-based automatic registration with 4DCT and adjusted by the radiotherapist based on soft tissue(mainly the primary tumor),setup error 2 was compared between 4DCBCT grayscale + manual registration and average intensity projection(AIP) of 4DCBCT grayscale + manual registration and setup error 3 was compared between 4DCBCT grayscale + manual registration and 3DCBCT grayscale + manual registration. Figure 1 shows the study flowchart.

The evaluation index included:

1. Dynamic change of tumor volume.
2. Dynamic analysis of tumor motion.
3. Stability of tumor centers.
4. 4D and 3D bone and gray value registration data.

## Results

Sixty-seven individuals who were diagnosed with lung cancer were enrolled. All of them had multiple 4D and 3D CBCT scans during the treatment. In total, 260 4D CBCT image datasets and 228 3D CBCT image datasets were collected.

### Analysis of tumor volume during treatment

The target area was outlined for 39 4D CBCT series, All tumors were delineated manually by two physicians with more than 5 years of clinical experience and reviewed by physicians on the same team with more than 20 years of clinical experience. The tumor volumes were calculated and compared.

**Fig. 2:**
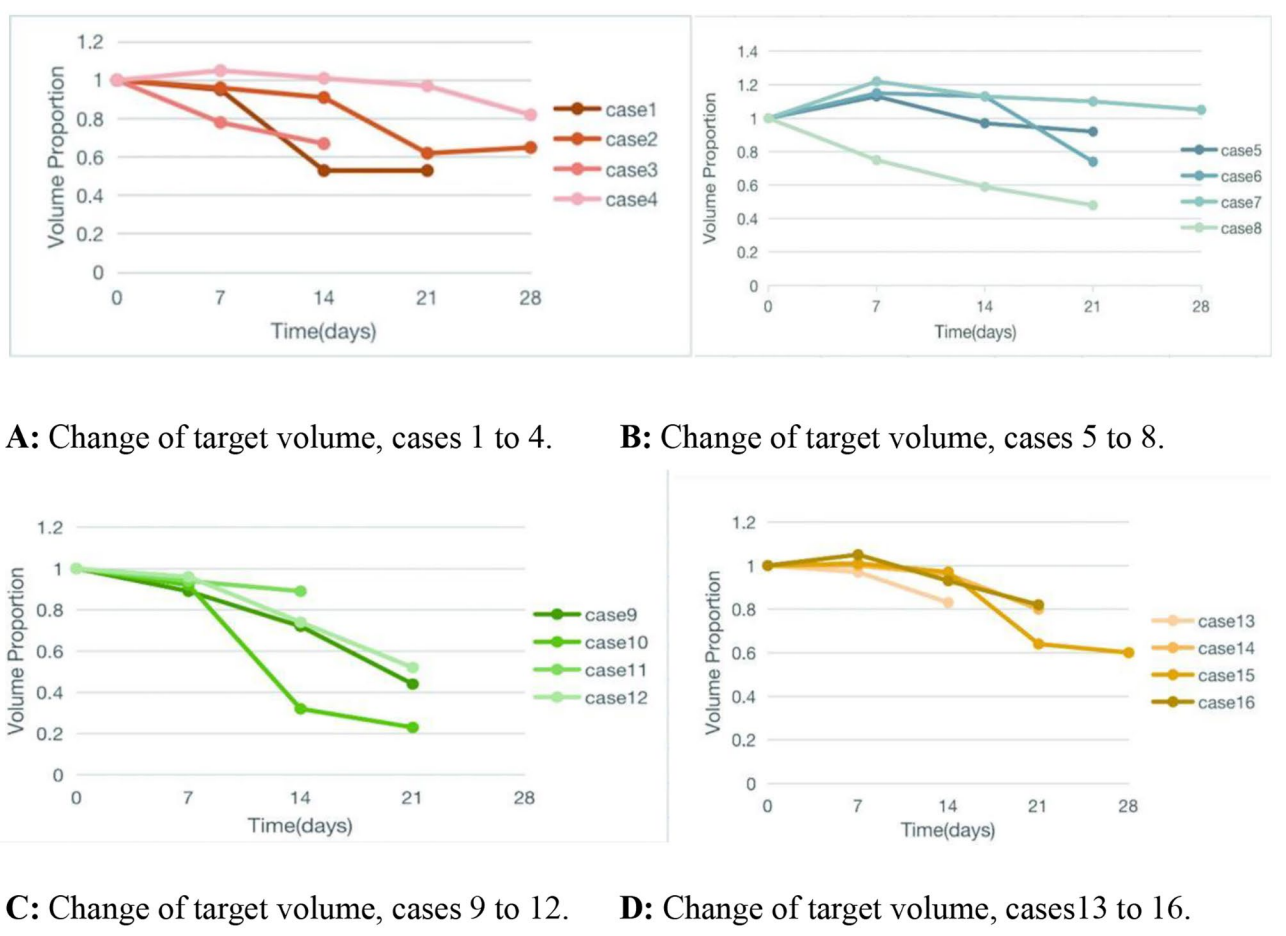
Dynamic change of target volume in 16 patients with tumor volume reduction. The median time to observed tumor volume response was 19 days (3 to 40 days)

1. The mean tumor volume in this group was 38.63 mL (0.44 to 285.78 mL). Tumor volume response (TVR) was observed in 16 (41.0%) cases during the treatment.
2. Tumor volume change curves for each 4D CBCT in the patients with TVR are shown in Fig. 2. The mean tumor volume was 57.06 mL (0.56 to 285.78 mL) in this group and the mean volume reduction was 41.70% (17–77%),The average volume reduction was 23.79mL(9.7mL-43.9mL). TVR was usually observed within the first 3 weeks, with a median time to response of 19 days (3 to 40 days).

### Analysis of tumor motion during treatment

1. In the initial 4D CBCT validation, tumor motion could be traced along three axes in 64 cases (Table 2), of which 13 (20%) were volume reduced and 3 (5%) were near the diaphragm. The X-axis is left-right,the Y-axis is superior-inferior, the Z-axis is anterior-posterior.
2. The mean value of the maximum difference in motion (the difference between the maximum and minimum motion) along the Y-axis was 0.24 cm (0.00 to 1.34 cm). In 16 cases (25%), the maximum difference was > 0.20 cm and the mean value of the maximum difference was 0.52 cm (0.22 to 1.34 cm) in these cases.
3. Tumor motion decreased in 10 cases and increased in 3 cases. The median time to maximum motion was 13 days after the first irradiation (5 to 40 days).
4. Three of the 6 lesions near the diaphragm demonstrated fluctuating tumor motion (0.28, 0.33, and 0.66 cm) with stable tumor volumes.

**Table 2:** Tumor motion along three axes (N = 64)

	N	Mean (cm)	Range (cm)
X-axis	20	0.33	0.12–0.80
Y-axis	64	0.91	0.13–2.40
≤ 0.20 cm	50	0.15	0.00–0.20
> 0.20 cm	14	0.52	0.22–1.34
Z-axis	18	0.31	0.11–0.55

Tumor motion was observed in all directions, mostly during the first 3 weeks of treatment; the most movement was measured along the Y-axis, in volume-reduced patients, and in the lesions near the diaphragm, which may show greater motion regardless of volume changes.

### Analysis of tumor center coordinates

The geometric center of the tumor was identified by outlining the target area in 39 4D CBCT images and determining the coordinates of the geometric center. The tumor center was calibrated by using 4D CBCT grayscale + manual registration data.

1. The average range of motion of geometric centers was 0.25 cm (0.03 to 1.44 cm) on the X-axis, 0.28 cm (0.03 to 2.50 cm) on the Y-axis, and 0.22 cm (0.01 to 0.73 cm) on the Z-axis.
2. Among 39 lesions, those with a range of motion < 0.5 mm were defined as centrally stable (N = 22) and those with a range of motion ≥ 2.5 mm as unstable (N = 17). The result of a univariate analysis of the mean tumor volume, tumor motion, and patient BMI in these two groups is shown in Table 3.

**Table 3:** Univariate analysis of factors influencing central stability (N = 39)

	Stable (N = 22)	Unstable (N = 17)	P
Average tumor volume	19.35 mL (0.44–85.12 mL)	63.60 mL (0.56–285.78 mL)	0.008
Range of motion	0.74 cm (0.00–2.38 cm)	1.03 cm (0.00–2.40 cm)	0.140
Body mass index	24.04 (20.00–27.76)	24.69 (15.85–28.73)	0.350

Tumor volume was found to be an independent factor affecting central stability (P = 0.008), and central stability was worst in patients with large volumes and a range of motion ≥ 2.5 mm. There was no statistical difference in range of tumor motion and body mass index between the two groups (P > 0.05)

### Registration with planning 4DCT and data analysis

#### Setup error1: Comparison of 4DCBCT bone-based registration and grayscale + manual registration

Patients (N = 67) were divided into a small error group (N = 39) and a large error group (N = 28); large error was present when the deviation between bone registration and 4D grayscale + manual registration was > 0.5 cm. Grayscale + manual registration was defined according to gray-based automatic registration with 4DCT and adjusted by the radiotherapist based on soft tissue(mainly the primary tumor).Univariate analysis of mean tumor volume, motion, and BMI was performed for both groups (Table 4).

**Table 4:** Factors influencing the error of four-dimensional cone beam computed tomography bone- based registration and grayscale + manual registration

	Small error group (N = 39)	Large error group (N = 28)	P
Average volume (mL)	43.30 (0.44–285.34)	40.98 (1.28–164.47)	> 0.05
Tumor Motion (cm)	0.74 (0.00–2.40)	1.03 (0.17–2.38)	0.044
Motion ≤ 0.5 cm (N = 21)	17 (43.5%)	4 (14.2%)	-
Motion > 0.5 cm (N = 46)	22 (56.5%)	24 (85.8%)	0.032
Body mass index	24.19 (15.85–20.05)	25.63 (22.49–32.53)	> 0.05

1. Tumor motion was the most influential factor in the difference between the two registration methods (P < 0.05); tumor motion was > 1 cm in the large error group.
2. Upon further analysis of the effect of tumor motion on registration errors, the partial volume off-target rate was 41.8% if based on bone registration only, 19.0% when the motion was ≤ 0.5 cm, and 52.2% when the motion was > 0.5 cm.

#### Setup error2: Comparison of 4DCBCT grayscale + manual registration and average intensity projection(AIP) of 4DCBCT grayscale + manual registration

Two sets of registration data were obtained: 4D grayscale + manual registration and AIP of 4DCBCT grayscale + manual registration. In 260 sets of 4D CBCT images, the margin of error was < 0.5 cm in three axes. There were only four occasions out of 260 (1.5%) where the registration verification process demonstrated and error of ≥ 0.30 cm (0.30 to 0.34 cm).The frequency of errors in each axis is shown in Table 5. There was no difference between the 4D CBCT grayscale + manual registration and AIP of 4DCBCT grayscale + manual registration.

**Table 5:** Registration errors in 4DCBCT grayscale + manual registration and AIP of 4DCBCT grayscale + manual registration

Registration error (cm)	X-axis (frequency, proportion)	Y-axis (frequency, proportion)	Z-axis (frequency, proportion)
0.00–0.10	203 (78.1%)	198 (76.3%)	198 (76.2%)
0.10–0.20	52 (20.0%)	62 (23.8%)	47 (18.1%)
>0.20	5 (1.9%)	0	15 (5.7)
Total	260 (100%)	260 (100%)	260 (100%)

#### Setup error3: Comparison of 4DCBCT grayscale + manual registration and 3DCBCT grayscale + manual registration

Because of the constraints of facility resources, patient treatment time, and radiation exposure, our trial used alternating 4D and 3D CBCT validation, which can reduce machine depreciation, decrease the chance of malfunction, reduce patient treatment time and the potential for movement during prolonged treatment, and limit the risk of excessive X-ray irradiation. However, setup errors, baseline differences, tumor volume, location, and motion changes may influence differences between the two methods of registration. Therefore, the 4D and 3D CBCT validation data were analyzed by excluding cases with significant tumor volume, motion changes, and unstable tumor centers. In the end, 21 cases were eligible for analysis. The bone registration data were subtracted from 4D and 3D CBCT grayscale + manual registration data to eliminate the influence of setup errors. When the two groups of data were compared, no difference was found in the 4D and 3D CBCT registration data of 14 cases on each axis, with 10 cases (71.4%) having tumors adjacent to the chest wall, mediastinum, or vertebral body. In 7 cases, there was a difference between the two kinds of registration (X-axis, 5 cases; Y-axis, 2 cases). Among these, the tumor was adjacent to the chest wall, mediastinum, or vertebral body in 3 cases (42.9%) (P = 0.296). Because of the relatively rigid anatomical reference, there was no difference between 4D CBCT and 3D CBCT registration when the tumor was adjacent to the chest wall, mediastinum, or vertebral body.

## Discussion

Accelerator-borne 3D CBCT is widely used to correct setup errors and guide precise radiotherapy because of its low radiation dose and fast scanning speed. 4D CBCT, which contains respiratory motion information, is the gold standard for positional correction in imaging-guided radiotherapy (IGRT) because it allows real-time monitoring of patient setup errors and target area motion. However, specific indications for 4D CBCT have not yet been clarified. This study explored the applicability of 4D CBCT by analyzing the changes in tumor volume, position, and respiratory motion during treatment. In addition, it explored the differences between bone and grayscale registration in 4D CBCT and 3D CBCT to provide additional clinical application schemes for the precise treatment of lung cancer.

4DCT has become the standard for outlining the intended treatment volume (ITV) throughout the respiratory cycle in precision radiotherapy for lung cancer. Mean intensity projection 4DCT images contain significantly more tumor motion information than 3D CBCT and 3DCT [5]. Wang et al. [6] compared ITV3DCBCT and ITV4DCT and found that the volume difference was 8% and Liu et al. [7] found that ITV3DCBCT was 11.8% smaller than ITV4DCT. Among the three methods, 4DCT was the most accurate in determining the tumor volume, followed by 3D CBCT, which had the largest error. However, the above methods did not consider respiratory motion and TVR during treatment. This study further explored the TVR by 4D CBCT during the treatment. TVR was observed in 41% of patients, with an average reduction of tumor volume of 41.7%; the response was observed within a median time of 19 days after starting radiotherapy. If 4D CBCT can be used to evaluate the TVR, especially at around the third week of treatment, it would allow for more precise second-course planning based on the extent of tumor reduction, reducing unnecessary irradiation of normal tissues and irradiation complications. In addition, 4D CBCT can indicate tumor regression during treatment and reflect the radiosensitivity, thus providing a reference for the individualized treatment mode.

Tumor motion is a hot topic in 4D CBCT research. Gottlieb et al. [8] used 4D CBCT to analyze baseline variability and motion patterns in 23 cases of lung cancer invading the mediastinum and found that 4D CBCT differed from localized 4DCT in the anterior-posterior direction before fractions 3, 10, and 20 (P < 0.05), suggesting that the motion of near-mediastinal tumors is prone to fluctuations with physiological conditions. Purdie et al. [9] compared the tumor motion trajectories of 4DCT and 4D CBCT images from 12 individuals with lung cancer diagnoses; the 4D CBCT motion trajectories matched those of 4DCT in 10 cases but showed significant deviations in the superior-inferior and anterior-posterior directions in 2. Sonke et al. [10] reported inter fractionated tumor motion on 4D CBCT in 56 individuals with lung cancer diagnoses; the range of motion range was mostly < 1.5 mm, with the most significant variation in the superior-inferior direction and in lower lobe lesions, and linear analysis revealed that the random error in baseline variation in three directions correlated with the mean tumor motion amplitude (P < 0.05). In this study, we confirmed that tumors could move in all directions, and also found that the maximum motion was along the Y-axis and in volume-reduced and diaphragm-adjacent lesions; volume reduction was most commonly observed in the third week of treatment. The tumors with greater motion were mostly those with TVR and those located near the diaphragm (0.28 to 0.66 cm). Similarly, previous studies suggest that tumors near the diaphragm or mediastinum or those with reduced volume might show greater motion in the Y-axis direction.

This study also found a statistical difference between tumor volume and central stability. Large-volume tumors (average tumor volume of 63 mL) have the greatest degree of central instability, i.e. the range of motion exceeded 2.5 mm. Small-volume tumors (average volume of 19 mL) had central motion < 2.5 mm. Tan et al. [11] included small lung cancers (maximum tumor diameter between 18 and 27 mm) in a comparison of 4D CBCT and 3D CBCT and found that setup errors were significant in all directions. Therefore, regardless of the tumor size, 4D CBCT can reflect the movement of the tumor center and reduce the uncertainty of tumor location caused by internal movement due to respiratory motion.

A comparison of 4D and 3D CBCT bone registration and manual grayscale registration reveals that when the tumor is more active, there is a 50% or higher probability of partial volume off-target when the registration is based solely on bone landmarks. If grayscale + manual registration is based on mean density projection, the effect is close to that of 4D CBCT grayscale + manual registration. There is no statistical difference between the registration data of 4D CBCT and 3D CBCT when the tumor is adjacent to the chest wall, mediastinum, or vertebral bodies because of the relative rigidity of the anatomical structures. Conversely, for lesions with isolated motion within the lung, especially for those with large ranges of motion, 4D CBCT may be more advantageous. Wang et al. [12] studied the difference between manual registration and gray registration, and found that gray registration could achieve better results than manual registration in the early stage of treatment, while the advantages of manual registration gradually emerged as radiotherapy progressed. Li et al. [13] selected the spine, spine + ITV, and lung for registration, with similar registration errors in the three groups. Schreibman et al. [14] explored the registration of full-time 4DCT and 4D CBCT in a case of liver cancer and demonstrated significant registration errors after bone registration, which were corrected after grayscale registration. Sweeney et al. [15] used 4DCT and 4D CBCT end-expiratory image registration errors as the gold standard (IG-4DCBCT) to compare 3D CBCT registration errors with IG-4DCBCT in 21 lung cancer patients and found that the difference between IG-4DCBCT and IG-3DCBCT was the largest (4.3 mm) in the superior-inferior direction; linear regression analysis indicated that the difference in registration between 4D CBCT and 3D CBCT gradually increased with increasing tumor motion. As per the findings of this study, when the tumor is adjacent to the chest wall, mediastinum, or vertebral body, because of its relatively rigid anatomical structure, accurate registration can be achieved by combining grayscale and manual 3D CBCT registration with bone registration. However, 4D CBCT with grayscale registration based on mean density projection is recommended when the tumor is located in an isolated region within the lung or is more mobile.

## Conclusion

Our results showed that patients treated with radiotherapy for lung cancer with intrapulmonary lesions have greater TVR and motion changes in the third week of treatment. As a result, 4D CBCT may be more advantageous for isolated lesions without reference to relative anatomical structures or proximity to the diaphragm, and grayscale registration based on mean density projection is feasible.

## Data Availability

The datasets used and/or analysed during the current study are available from the corresponding author on reasonable request.

## Notes

The original online version of this article was revised: the second author ‘Xu Yang’ should be co-first author with ‘Muyasha Abulimiti’.
