# Correction: Application of four-dimensional cone beam computed tomography in lung cancer radiotherapy

**DOI:** 10.1186/s13014-023-02280-x

**Published:** 2023-07-12

**Authors:** Muyasha Abulimiti, Xu Yang, Minghui Li, Fukui Huan, Yanxin Zhang, Liang Jun

**Affiliations:** 1 National Cancer Center, National Clinical Research Center for Cancer/Cancer Hospital & Shenzhen Hospital, Chinese Academy of Medical Sciences and Peking Union Medical College, Shenzhen, 518116 China; 2 Department of Radiation Oncology, National Clinical Research Center for Cancer/Cancer Hospital, National Cancer Center, Chinese Academy of Medical Sciences and Peking Union Medical College, Beijing, 100021 China

**Radiation Oncology (2023) 18:69**

10.1186/s13014-023-02259-8.

After publication of this article [1], the authors reported that the second author ‘Xu Yang’ should be co-first author with ‘Muyasha Abulimiti’.

The original article [1] has been corrected.
